# Cerebral Autoregulation, Brain Injury, and the Transitioning Premature Infant

**DOI:** 10.3389/fped.2017.00064

**Published:** 2017-04-03

**Authors:** Zachary A. Vesoulis, Amit M. Mathur

**Affiliations:** ^1^Division of Newborn Medicine, Edward Mallinckrodt Department of Pediatrics, Washington University School of Medicine, St. Louis, MO, USA

**Keywords:** autoregulation, near-infrared spectroscopy, intraventricular hemorrhage, white matter injury, brain injury, prematurity

## Abstract

Improvements in clinical management of the preterm infant have reduced the rates of the two most common forms of brain injury, such as severe intraventricular hemorrhage and white matter injury, both of which are contributory factors in the development of cerebral palsy. Nonetheless, they remain a persistent challenge and are associated with a significant increase in the risk of adverse neurodevelopment outcomes. Repeated episodes of ischemia–reperfusion represent a common pathway for both forms of injury, arising from discordance between systemic blood flow and the innate regulation of cerebral blood flow in the germinal matrix and periventricular white matter. Nevertheless, establishing firm hemodynamic boundaries, as a part of neuroprotective strategy, has challenged researchers. Existing measures either demonstrate inconsistent relationships with injury, as in the case of mean arterial blood pressure, or are not feasible for long-term monitoring, such as cardiac output estimated by echocardiography. These challenges have led some researchers to focus on the mechanisms that control blood flow to the brain, known as cerebrovascular autoregulation. Historically, the function of the cerebrovascular autoregulatory system has been difficult to quantify; however, the evolution of bedside monitoring devices, particularly near-infrared spectroscopy, has enabled new insights into these mechanisms and how impairment of blood flow regulation may contribute to catastrophic injury. In this review, we first seek to examine how technological advancement has changed the assessment of cerebrovascular autoregulation in premature infants. Next, we explore how clinical factors, including hypotension, vasoactive medications, acute and chronic hypoxia, and ventilation, alter the hemodynamic state of the preterm infant. Additionally, we examine how developmentally linked or acquired dysfunction in cerebral autoregulation contributes to preterm brain injury. In conclusion, we address exciting new approaches to the measurement of autoregulation and discuss the feasibility of translation to the bedside.

## Introduction

Premature infants weighing less than 1,500 g at birth are frequently affected by two specific forms of brain injury, such as intraventricular hemorrhage (IVH) and white matter injury (WMI). In the last 30 years, the incidence of IVH has fallen from nearly 50% (1) to 25% (2) as a result of antenatal steroid administration, improved ventilator strategies, and improved obstetrical practices. In contrast, the incidence of WMI has remained essentially unchanged, affecting approximately 10% of infants less than 1,500 g (3). Both forms of injury are linked to the development life-long neurodevelopmental impairment, including cerebral palsy (4, 5).

Although there long have been defined associations between clinical factors (e.g., pneumothorax, need for cardiopulmonary resuscitation, sepsis, and necrotizing enterocolitis) and both forms of brain injury (5), the precise underlying mechanisms have remained less clear, despite the efficacy of some neuroprotective strategies. Given histologic evidence (6) suggesting the injury arises from discordance between systemic and cerebral blood flow (CBF) in the germinal matrix and periventricular white matter, a primary target for understanding the mechanism of brain injury, should be based in the investigation of perfusion. Current methods for assessing the adequacy of cardiac output to support proper organ system are typically imprecise, difficult to perform, are not feasible for longitudinal monitoring, or lack specificity to cerebral circulation.

Heart rate and capillary refill time remain key parts of the primary hemodynamic assessment. As neonates lack the ability to alter stroke volume, tachycardia is the primary means for increasing cardiac output, yet frequent confounding factors such as pain/agitation and caffeine citrate administration can confound the heart-rate assessment (7). Capillary refill can also be a very specific indicator of hemodynamic compromise; however, it lacks sensitivity (approximately 35%) needed for consistent assessment (8). Echocardiography remains the “gold standard” of evaluating volume status and vascular tone (preload and afterload), essential for estimate of cardiac output; however, it requires the skills of a cardiac sonographer and an expert interpreter and is impractical for longitudinal monitoring. Blood flow through the superior vena cava, a measure of volume status, can also be measured by echocardiography (9) but again faces the challenge of longitudinal data capture. A similar measure of right ventricular filling can be evaluated by measurement of the central venous pressure *via* an umbilical venous catheter; however, there are significant challenges in interpretation of these values as there is a broad range of normal values, hampering discrimination between normal and pathologic states (10).

Over the last 10 years, investigation has shifted from examination of systemic blood flow to detailed investigation of patterns of CBF. This shift has been enabled by new technology, driving deeper investigation into the regulation of CBF in preterm infants, finding significant impairment of autoregulation as a major contributing factor to the development of IVH. Additionally, new strategies for identifying and classifying WMI on MRI have changed our views of the link between WMI and neurodevelopmental outcome (11). Cutting-edge techniques to identify WMI using simple, bedside monitoring provide an opportunity for earlier diagnosis and counseling of the family.

Taken together, technological advancements now provide a window, for the first time, into the detailed interplay of cerebrovascular regulation, ischemia–reperfusion, and the development of brain injury in preterm infants. These advances offer the potential for early detection of impending brain injury, allowing the opportunity for intervention with the aim of prevention or minimization of injury.

In this review, we seek to discuss advances in the field of brain monitoring, with a focus on hemodynamics, as related to the development of preterm brain injury. We explore new knowledge about the role clinical factors and management play in providing additional neuroprotection or contributing to worsened injury. Finally, we discuss future developments in the field including the potential for new clinical management strategies, which incorporate real-time, multimodality bedside monitoring in the NICU.

## Mechanisms of Injury—IVH

During fetal development, neurons and glial cells arise from the germinal matrix and, sub-ventricular zone and migrate outwards toward the cortex. This process requires rich vascular support from a dense capillary bed within the germinal matrix. Unlike mature blood vessels, those of the germinal matrix are thin-walled, lack pericytes and have limited glial fibers, making them extremely fragile. This capillary bed drains into the terminal vein before entering the internal cerebral vein (5).

Two postulations have emerged as to the pathogenesis of IVH. In one theory, significant fluctuations in arterial blood pressure, the result of sepsis, noxious stimuli, fluid boluses, or inotrope drugs, overwhelm the fragile capillary bed and lead to the bursting of the vessels. Alternatively, obstruction of the venous system, whether by pneumothorax impeding venous return, ventilator asynchrony or simply a change in head position caused an increase in hydrostatic pressure, again leads to the rupture of vessels in the capillary bed (5).

Both mechanisms likely provide contributory components, with cycles of ischemia and reperfusion further weakening the vascular structures until the blood vessels can no longer tolerate the fluctuations and burst.

## Mechanisms of Injury—WMI

Investigation into the underlying pathophysiology of WMI has revealed a link between three predisposing factors (disturbances in cerebral oxygenation, infection, and inflammation) and a developmental vulnerability of the white matter between 23 and 32 weeks of development (5, 12). Premyelinating oligodendrocyte progenitor cells (pre-OL), a subtype of glial cells that proliferate in waves during the second trimester, are found in high densities in the white matter and exhibit a maturation-dependent vulnerability to oxidative stress (5, 13). This vulnerability can be intrinsic, caused by excessive glutathione depletion from oxidative stress or extrinsic, caused by glutamate receptor-mediated toxicity, the result of glutamate release by adjacent dying cells (14). Either mechanism sets up a cascade of events, leading to loss of pre-OLs and damage to surrounding white matter (15–17).

The premature brain is vulnerable to oxidative stress from hypoxic–ischemic injury as a result of the confluence of three key risk factors: heart-rate dependent cardiac output, the immature vascular supply, and disturbances in vascular autoregulation. First, continued adequate cardiac output in premature infants is tenuous; given that stroke volume is relatively fixed, cardiac output is dependent on heart rate (13). Any disturbance in the heart rate [e.g., apnea of prematurity, with resulting bradycardia (18)] sets up the possibility of ischemia. Second, the white matter, particularly in the periventricular zone, is supplied by the long penetrating arteries, which first arise around 24 weeks of gestation but maintain remarkably low levels of blood flow, with minimal collateralization, until 30–32 weeks of gestation (6, 19). Finally, there is evidence of an impaired cerebrovascular autoregulatory system, suggesting that the preterm brain has limited capacity to regulate blood flow when oxygen saturation or cardiac output is reduced (20, 21). Repeated ischemic episodes associated with any of these mechanisms will generate an inflammatory response, thus increasing the metabolic demand, utilizing a greater fraction of delivered oxygen, and potentiating further injury during the next ischemic episode.

## Review of Cerebrovascular Autoregulatory System

**Figure 1:**
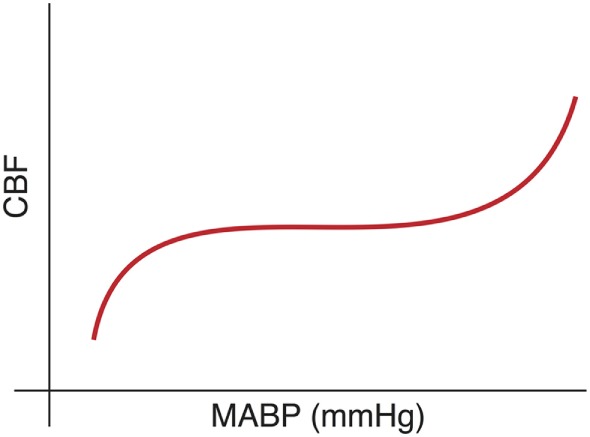
**Cerebral blood flow (CBF) is conceptualized as a sigmoidal curve with stable blood flow across a range of “normal” blood pressure and impairment at either extreme**.

The cerebrovascular autoregulatory system is a physiologic mechanism, which functions to maintain constant and stable CBF. The classic depiction of this system is a sigmoidal curve (Figure 1) with stable CBF over a range of normal blood pressures and unstable CBF when the blood pressure is outside of this range. The work of Lou (22) and Ment (23) demonstrated the failure of the preterm cerebral vasculature to maintain uniform cerebral perfusion over a range of systemic blood pressures, a phenomena termed “pressure-passive circulation.”

A complementary view of blood flow regulation was initially proposed in the nineteenth century by Siegmund Mayer when he observed spontaneous rhythmic oscillations in blood vessel diameter, with a periodicity of 10 s (0.1 Hz), in the wings of bats. Recent computational techniques have allowed greater exploration of this dynamic model, with data suggesting that the autoregulatory system attenuates the effect of low-frequency fluctuations in blood pressure, effectively functioning as a high-pass filter (24, 25). These oscillations, now called “Mayer waves,” arise from changes in the vasomotor tone of arterial blood vessels throughout the body and are likely driven by the autonomic nervous system.

Oscillations in blood pressure can divided into three categories based on their periodicity—high frequency (HF, >0.5 Hz), low frequency (LF, 0.05–0.5 Hz), and very-low frequency (VLF, <0.05 Hz). HF fluctuations are driven by the respiratory cycle (and thus are relatively fixed). LF and VLF variability represent the composite influence of autonomic (26), myogenic (27, 28), and cellular (29) control mechanisms. Crucially, the intensity of response of these control mechanisms is modulated by circulating catecholamines (30), vasoactive intestinal peptides (31), nitric oxide (26, 32, 33), hypovolemia (34), and the renin–angiotensin system (35). Cerebrovascular autoregulatory system failure results in unstable CBF, generating the cycle of ischemia–reperfusion, which drives the mechanisms of preterm brain injury.

## Overview of Methods for Quantifying the Autoregulatory System

Methods for quantifying the state of the autoregulatory system have evolved over time in conjunction with advances in technology. Reliably testing the function of the cerebral autoregulatory system in the preterm population, however, has proven to be a difficult task, largely due to the broad diversity in neurophysiologic development, technical challenges associated with capturing data, the lack of a clear definition for hypotension (36), and lack of a standardized analysis methodology. The earliest approaches that used PET, transcranial Doppler ultrasound, or ^133^Xe clearance provide a dynamic view of the cerebral vasculature. While effective, they are not feasible for longitudinal monitoring due to concerns about radiation exposure, increased temperature in the target tissue (37), and the use of invasive delivery of radioactive isotopes, respectively. Furthermore, they provide only semiquantitative information about autoregulation.

**Figure 2:**
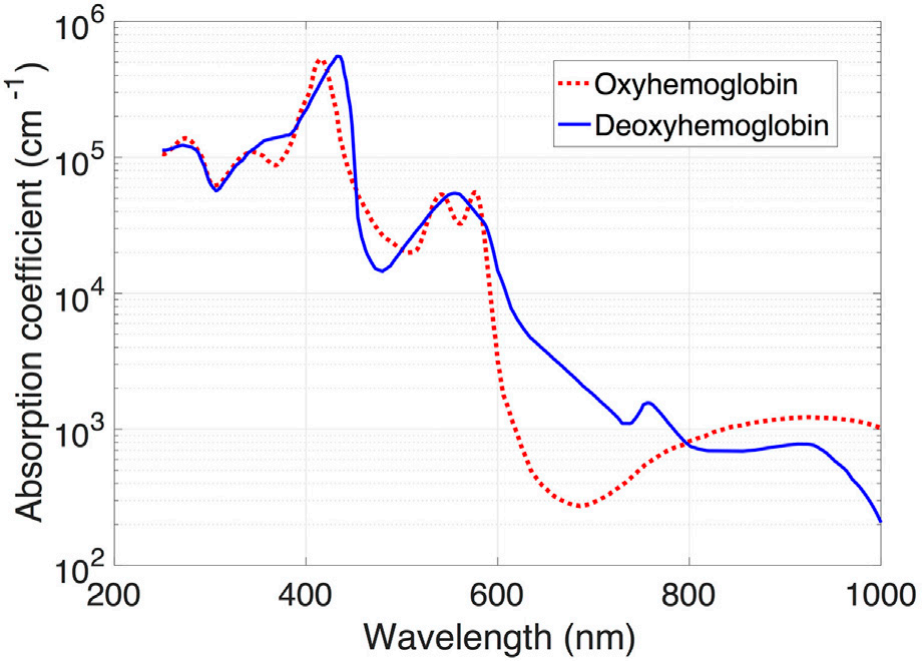
**Absorption spectra of light for oxy- and deoxyhemoglobin**. Note the decreased absorption by oxyhemoglobin in the infrared band (approximately 700 nm).

The use of near-infrared spectroscopy (NIRS) to quantify cerebral flow represents a revolutionary step in obtaining longitudinal hemodynamic information and comes with a dramatically improved safety profile in the vulnerable preterm neonate. The mixed-venous oxygen saturation of hemoglobin in a tissue can be estimated using NIRS, a technique that uses the difference in absorption of near-infrared light by oxy- and deoxyhemoglobin to detect the relative concentrations of each compound (Figure 2). Given that only 30% of blood is intra-arterial at any given time, the measured values represent a 30/70 arterial/venous-weighted estimate of oxygen saturation. NIRS has been used extensively in the neonatal population, with recent publication of a reference dataset in a cohort of nearly 1,000 infants, suggesting a typical cerebral saturation value of approximately 65% (95% CI 55–75) (38).

Venous oxygenation is influenced by many parameters, including cardiac output, blood pressure, oxygen content of the blood, pH, and metabolic demand by tissues. These properties are highly advantageous to the study of regional tissue oxygenation, as these measurements reflect the amount of oxygen available to and consumed by the target of interest. This stands in contrast to arterial saturation measured by pulse oximetry (SpO_2_), which initially may remain at or near 100% despite significant ischemia in regions of the body. Although NIRS directly measures tissue oxygenation, it is highly correlated with measures of blood flow made by other measures (39), making it an attractive proxy for more invasive alternatives.

While other factors, such as method of respiratory support (non-invasive, invasive, and high-frequency oscillatory ventilation), do not directly affect cerebral oxygenation, NIRS measurements have been used in a variety of studies to optimize respiratory support including prediction of extubation failure (40), an alternative to pulse oximetry for titration of supplemental oxygen (41), or early detection of hypercapnia (42).

Even with advancing technology, the function of the autoregulatory system cannot be measured directly. Instead, techniques have evolved, which derive function by measuring how an input signal (arterial blood pressure) is shaped and altered into the output (CBF). Optimal autoregulation would maintain steady-state CBF, despite changes in systemic blood pressure, while impaired autoregulation would be manifested by a high degree of correlation between changes in the blood pressure and changes in CBF. Two different approaches to utilizing NIRS data to quantify the autoregulatory system have emerged in the literature.

**Figure 3:**
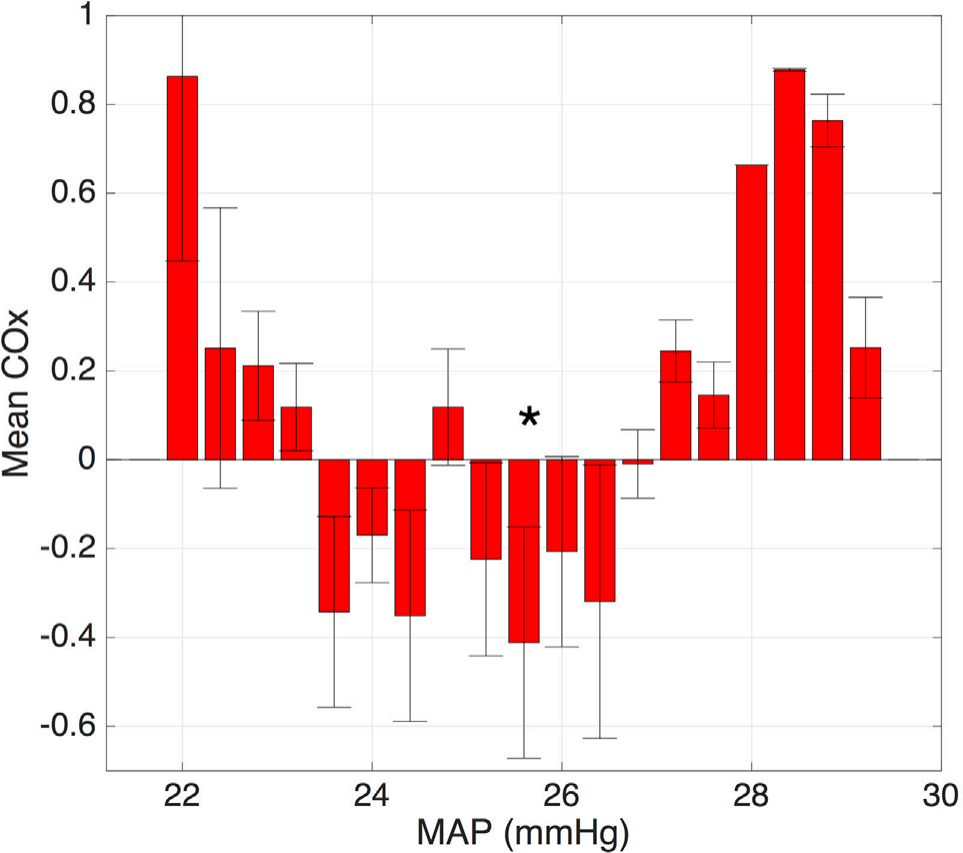
**Plot of the average correlation between the blood pressure and cerebral oxygenation**. Note the lack of correlation over the range of “normal” blood pressure and increased correlation at the extreme values, representing loss of autoregulation. The optimal MAP is denoted with an asterisk.

One approach utilizes *time-domain analysis*, examining the correlation between the measured values of blood pressure and cerebral saturations. This approach has been extensively studied and optimized by Lee et al. (43–50). In this approach, the correlation between short segments of blood pressure and NIRS data is continuously calculated. The mean correlation value is then calculated for each possible blood pressure value and sorted into “bins.” The resulting output (Figure 3) is reminiscent of the autoregulation curve shown in Figure 2 with high correlation values at extremes of blood pressure and 0 or negative correlation values in the “normal” range of blood pressure (Figure 3).

An alternate approach utilizes *frequency-domain analysis*, examining the correlation between low-frequency oscillations in the blood pressure and those in CBF. Several different research groups have utilized spectral coherence, a statistical method for comparing the relationship between two signals (20, 51, 52) with resulting values between 0 (no coherence) and 1 (perfect coherence). By setting a threshold of 0.5, researchers were able to calculate the proportion of time that infants were “pressure-passive.” This approach is advantageous in that it is able to measure autoregulation as a dynamic system; however, the nature of frequency-domain analysis demands a high degree of precision in time synchronization of data capture (53). This pressure-passive pattern has been documented to occur in 10–20% of measured epochs in VPT infants, and there is a suggestion that it is found in association with brain injury (20, 22, 54).

**Figure 4:**
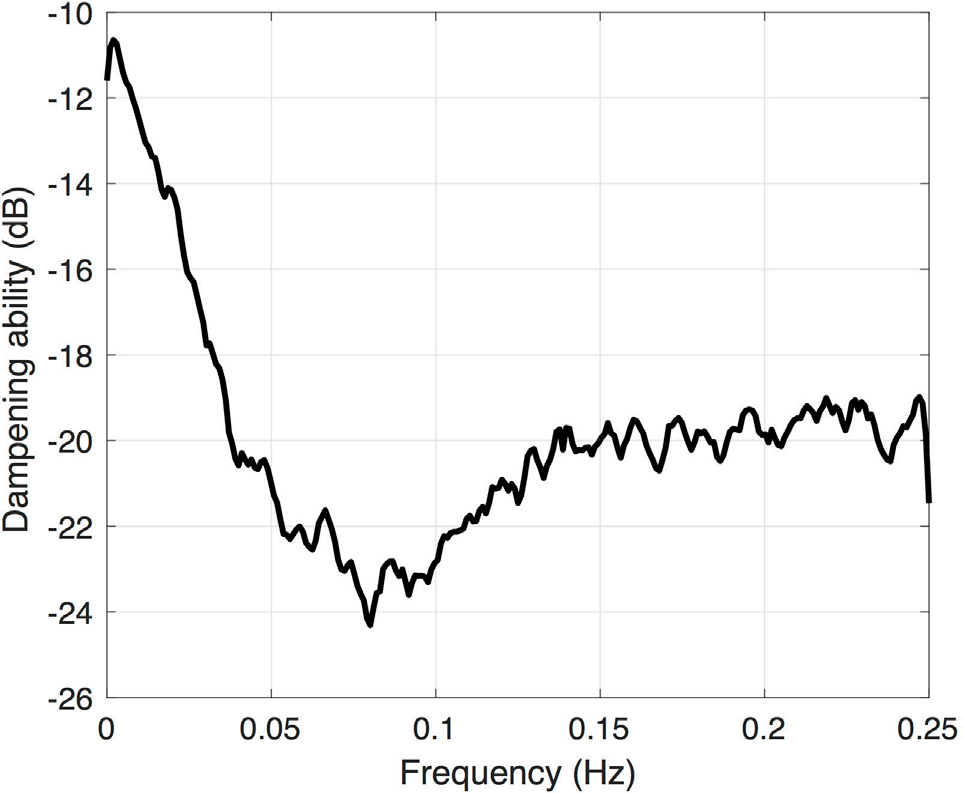
**Transfer function gain coefficient calculated between the mean arterial blood pressure and cerebral oxygenation**. Note the increased dampening (more negative value) around 0.1 Hz.

Another frequency-domain approach utilizes *transfer function analysis*, which derives the function of the autoregulatory system by examining how well the autoregulatory system is able to dampen oscillations in systemic blood pressure. Rather than using pre-defined thresholds, this technique utilizes natural low-frequency fluctuations in both the blood pressure and CBF. The resulting output resembles a band pass filter (Figure 4), with dampening of low-frequency oscillations around the Mayer wave frequency (0.1 Hz). There is also a developmentally linked component to this dampening, with increased dampening found in infants of increasing gestational age (21).

## Link Between Dysfunction in Autoregulation and Brain Injury

There are many interconnected modulators of vascular tone, governed by a mix of local and central mechanisms, each of which contributes to the overall function of autoregulation. The most potent endogenous factors include the partial pressures of carbon dioxide and oxygen, and the autonomic nervous system. CO_2_ exerts a direct vasodilatory effect, leading to excessive oxygenation, even without supplemental oxygen delivery, if left unchecked (41) with small increases in CO_2_ leading to profound increases in CBF. Oxygen, or more specifically hypoxia, provides a similar response; the degree of vasodilation is inversely correlated with the partial pressure of oxygen. Acute hypoxia can lead to dramatic increasing in vasodilation, and thus CBF (55). The autonomic nervous also plays an important role, with counterbalancing release of nitric oxide and catecholamines in response to sympathetic and parasympathetic stimulation (56).

As a result of this constellation of factors, maintenance of stable CBF in the vulnerable preterm infant is tenuous. Many complicating clinical factors have been linked to causing or worsening the dysfunction of the autoregulatory system including hypotension (20), hypoxic-ischemia (57, 58), seizures (59), inotropic medications (60), and possibly even IVH itself (21).

Brain injury, in the form of hypoxia–ischemia–reperfusion, is the common end result of the failure of the autoregulatory system, regardless of the contributing factors. Early in the course of hospitalization, repeated episodes of ischemia and reperfusion likely lead to weakening and then rupture of the fragile germinal matrix vessels. Indeed, impaired autoregulation has been clearly identified in infants with IVH (20, 21). This mechanism is also a likely contributing factor to WMI, where repeated episodes of ischemia, from a mismatch of perfusion and metabolic demand, accumulate in the periventricular white matter, a region with relatively limited vascular supply at this stage of development. Repeated events undoubtedly lead to neuronal death and start the cascade of events leading to gliosis and fiber loss. The threshold at which either form of brain injury occurs is not known at this time.

## The Next Step—Translation to the Bedside

While much has been learned about the function of the cerebrovascular autoregulatory system in premature infants and its role in the pathogenesis of brain injury, there are a number of key factors in translation which remain to be solved before a bedside “autoregulation monitor” can be implemented.

Primary among them is developing a consensus on the approach to quantification. Although each of the described approaches converges on essentially the same results, the approaches are quite disparate. The ideal system would be readily reproducible across patient populations and clinical confounders. It is possible that the best approach is, in fact, a hybrid of above methods, each contributing an important component of knowledge.

Another key factor is the source of data for quantification. All of the current approaches require the use of NIRS and invasive arterial blood pressure monitoring. These sources of information allow one to readily model the input and output sides of autoregulation, but obtaining these data is costly and may not be feasible or practical. Future investigation should focus on other sources of data (e.g., ECG and pulse oximetry), which may provide some of the same data, without the need for separate, invasive equipment.

In addition to the hemodynamic data provided by the approaches outlined above, a complete brain monitoring system should also include functional measures. This could most easily be accomplished by the use of amplitude integrated EEG (aEEG), which is able to provide information about the general background activity of the brain as well as detection of seizures, a well-described methodology in the neonatal population (61). This information would allow real-time feedback as the effects of impaired autoregulation and may help to better discriminate between transient asymptomatic aberrations or more consequential ones (62).

Finally, and most crucially, techniques for manipulating the autoregulatory system through hemodynamic intervention (e.g., the targeted use of inotrope medications) while guided by instantaneous measurement will allow for the advancement of neuroprotective strategies. To date, studies have focused on *ex post facto* examination of recorded data. Developing a real-time autoregulation monitor, along with methods for responding to the readout, will be essential for this to be part of a successful neuroprotection strategy (63).

## Conclusion

In conclusion, the incidence of the two most common forms of brain injury in preterm infants, such as IVH and white matter, remains distressingly high, and current strategies for neuroprotection do not extend beyond acknowledgment of static demographic risk factors. One potential target is augmenting the function of the cardiovascular system through anti-hypotensive therapies, including inotropes and fluid resuscitation, yet there is a lack of consistent data as to the lower limit of arterial blood pressure, which confers an increased risk of brain injury. A bedside monitor capable of demonstrating the function (dysfunction) of the autoregulatory system, aided with integration of a secondary source of information such as aEEG, will allow for personalized blood pressure management, avoiding overtreatment of “hypotension” in infants with intact autoregulation and enhancing recognition of poor cerebrovascular health in infants with “normal” blood pressures.

## Author Contributions

ZV participated in the conceptualization of the manuscript, wrote the initial draft, and approved the final version as submitted. AM participated in the conceptualization of the manuscript, provided a critical review of the manuscript, and approved the final version as submitted.

## Conflict of Interest Statement

The authors have no financial ties or potential/perceived conflicts of interest with any of the products or manufacturers described in this manuscript.
